# EUROPEAN OLDER ADULTS FRAIL: FINDINGS FROM THE SURVEY OF HEALTH, AGEING AND RETIREMENT IN EUROPE (SHARE)

**DOI:** 10.1093/geroni/igz038.2515

**Published:** 2019-11-08

**Authors:** Giulia Manfredi, Luís Midão, Constança Paúl, Clara Cena, Mafalda Duarte, Elísio Costa

**Affiliations:** 1 Department of Pharmaceutical Science and Technology, Via P. Giuria 9, I-10125 Turin, Italy; 2 ICBAS - Abel Salazar Institute of Biomedical Sciences, University of Porto, Porto, Portugal, Porto, Portugal; 3 Instituto de Ciências Biomédicas Abel Salazar, Universidade do Porto, Porto, Portugal; 4 Department of Pharmaceutical Science and Technology, Via P. Giuria 9, I-10125 Turin, Italy, Turin, Italy; 5 ISAVE – Superior Institute of Health, Amares, Braga, Portugal, ISAVE – Superior Institute of Health, Braga, Portugal; 6 UCIBIO/REQUIMTE, PORTO4AGEING - Competences Centre on Active and Healthy Ageing of the University of Porto, Faculty of Pharmacy of the University of Porto, Porto, Portugal, UCIBIO/REQUIMTE, Portugal

## Abstract

Frailty is a geriatric multidimensional syndrome whose signs and symptoms are predictors of increased vulnerability to minor stress events and risk of adverse outcomes such as falls, fractures, hospitalisation, disability and death. In this work, we aimed to update the data of frailty status in European community dwelling population, based on the latest data released (wave 6) of SHARE database, and to study the impact of each criterion on frailty assessment. Frailty status was assessed applying a version of the Fried Phenotype operationalised for SHARE. We included all participants who answered all the questions used in a frailty assessment and who disclosed their gender and, further, whose age was 50 or more. Our final sample was 60816 individuals. Of these, the mean age was 67.45 ± 9.71 years; 38497 (56.4%) were female. The overall prevalence of pre-frailty was 42.9% (ranging from 34.0% in Austria to 52.8% in Estonia) and frailty was 7.7% (ranging from 3.0% in Switzerland to 15.6% in Portugal). Pre-frailty and frailty prevalence increased along age and were more frequent among women. Regarding the five criteria considered on frailty assessment, exhaustion seems to be the criterion that contributes most to frailty status, followed by low activity, weakness, loss of appetite and slowness. With this work, we demonstrated that more than 50% of the 50+ European population are pre-frail/frail, which must be considered when designing interventions to reduce/postpone/mitigate the progression of this condition, reducing the burden associated with it.

